# Enhancing the Anti-Aging Potential of Green Tea Extracts Through Liquid-State Fermentation with *Aspergillus niger* RAF106

**DOI:** 10.3390/foods14203548

**Published:** 2025-10-18

**Authors:** Yuju Liu, Xiao Zhang, Xingbing Liu, Ruixuan Li, Ximiao Yang, Zhenlin Liao, Xiang Fang, Jie Wang

**Affiliations:** College of Food Science, South China Agricultural University, Guangzhou 510642, China; liuyuju@163.com (Y.L.); zhangxiaohuge@163.com (X.Z.); 13411135745@163.com (X.L.); liruixuan0410@163.com (R.L.); yxm202220220@163.com (X.Y.); larryliao@scau.edu.cn (Z.L.); fxiang@scau.edu.cn (X.F.)

**Keywords:** anti-aging, *Aspergillus niger*, green tea extract, metabolites, liquid-state fermentation

## Abstract

Microbial fermentation diversely modulates the bioactivity of green tea extracts (GTE), but its effects on anti-aging potential remain under-explored. This study investigated the effects of liquid-state fermentation by *Aspergillus niger* RAF106 on the anti-aging properties of GTE from Biluochun and identified its longevity-promoting metabolites. The unfermented GTE used herein showed no or limited effects, but the four-day fermented tea extracts (GTE-A4) significantly extended the mean lifespan in *Caenorhabditis elegans*, enhanced motility and stress resistance, and improved mitochondrial function and antioxidant properties, while reducing lipid accumulation and oxidative damage. The pro-longevity effect depended on insulin/IGF-1, MAPK, and p53 pathways and required transcription factors DAF-16 and HSF-1. Fermentation periods shorter or longer than 4 days led to reduced efficacy. Fermentation with RAF106 dynamically altered chemical composition and induced the enrichment of various longevity-promoting metabolites in GTE-A4, including proanthocyanidin A2, aromadendrin, and dalbergioidin—all newly identified as anti-aging agents. These findings demonstrate that RAF106 fermentation improves the anti-aging potential of green tea and provides a scientific basis for using precision fermentation to develop advanced anti-aging functional ingredients from tea extracts.

## 1. Introduction

Aging, a ubiquitous, complex, and progressive process, leads to a decline in physiological integrity and function, and is associated with various chronic diseases, including neurodegenerative diseases, cardiovascular diseases, and cancer. Therefore, the rapid increase in the global aging population has imposed a significant burden on healthcare systems and economies, making interventions to delay aging a topic of widespread interest [1,2,3]. Dietary intervention is a recognized strategy for delaying aging, and functional foods have become an important choice because of their efficacy and convenience. Studies have shown that functional foods such as polysaccharides, oligosaccharides, probiotics, and dietary polyphenols have significant anti-aging effects in different models [4]. Tea, the second most widely consumed beverage after water, is a rich source of polyphenolic compounds and has been extensively studied for its health benefits, including antioxidant, anti-obesity, anti-arteriosclerotic, anti-carcinogenic, and antibacterial activities [5,6,7]. Recently, tea consumption could be a promising strategy to promote healthy longevity and delay aging [8]. Notably, green tea, white tea, yellow tea, oolong tea, black tea, and dark tea could extend lifespan and improve health span through multiple pathways, such as the insulin/IGF-1 signaling (IIS) pathway, the NAD^+^/SIR-2.1 pathway, and dietary restriction [8,9,10]. Moreover, the anti-aging effects of teas depended on cultivars. Among 11 cultivars of green tea, Yiwu, Bulanshan, and Xigui significantly prolong lifespan, while Shuchazao and Hekai failed to prolong lifespan [10].

Fermentation can improve the sensory properties, nutritional value, bioavailability, and bioactivity of food products, making it a promising strategy for developing functional foods [11]. Microbial fermentation could affect the physiological functions of tea extracts or tea leaves in a strain-dependent manner. For example, fermentation with *Lactobacillus plantarum* or *Lactobacillus brevis* altered phenolic composition, elevated total phenolic content, and increased cellular antioxidant activity in green tea extracts and black tea extracts [12], and fermentation with *Aspergillus oryzae* decreased flavonoid content and increased antioxidant activity in green tea extracts [13], while fermentation with *Trichoderma* spp. reduced the phenolic and flavonoid content and antioxidant activities in Assam tea leaves [14]. *Cordyceps militaris* fermentation altered the flavor and chemical profiles of Lu’an GuaPian green tea with fat-lowering and anti-aging activities [15]. *Aspergillus niger*, a fungus generally recognized as safe for use in the manufacture of fermented foods and biotechnology products, acts as one of the dominant microorganisms in the fermentation process of Pu-erh tea and Tibetan tea to produce the bioactive and flavor compounds through enzymatic reactions mediated by tannase, laccase, peroxidase, and so on [16,17,18,19]. However, the anti-aging effects of *A. niger*-fermented teas remain under-explored.

Previously, *A. niger* RAF106, a beneficial and safe fungus isolated from Pu-erh tea, could detoxify aflatoxin B1 and biotransform ester-catechins [20,21,22,23]. Moreover, solid-state fermentation with *A. niger* RAF106 improved the flavor profile in summer green tea and the Yunnan large-leaf variety of sun-dried green tea [16,24]. However, systematic investigations into the effect of *A. niger* RAF106 on the chemical composition of green tea during liquid-state fermentation, a process which can shorten fermentation time, minimize microbial contamination, and pave the way for a more automated production line [25], are lacking, as well as the investigations into its anti-aging efficacy. Therefore, this study aimed to investigate the changes in metabolic profile using untargeted metabolomics and in anti-aging effects using *Caenorhabditis elegans*, a primary model organism for aging and the discovery of natural products for healthy aging [26], after liquid-state fermentation with *A. niger* RAF106 in green tea extracts. Moreover, the underlying anti-aging mechanisms of RAF106-fermented tea extracts and the potential metabolites were unveiled. Collectively, the findings highlighted that fermentation with RAF106 can improve the anti-aging effects of green tea extracts, and RAF106-fermented tea extracts can potentially act as a novel intervention for aging delay and health promotion.

## 2. Materials and Methods

### 2.1. Materials

*Escherichia coli* OP50 (OP50) was purchased from the *Caenorhabditis* Genetics Center (CGC) and incubated in Luria–Bertani (LB) broth at 37 °C with shaking. Pu-erh tea-derived *A. niger* RAF106 (CGMCC No.9608) was cultivated in potato dextrose agar (PDA) at 30 °C to produce conidia [20].

The wild-type *C. elegans* N2 and mutants were purchased from CGC and cultivated on modified nematode growth medium (NGM) in 6-cm petri dishes supplemented with OP50 cells, an international standard food for *C. elegans*. Eggs were harvested from egg-bearing worms treated with sodium hypochlorite–sodium hydroxide solution and fed with OP50 cells.

Sinapaldehyde, proanthocyanindin A2, prodelphinidin A1, gallocatechin, neoisoastilbin, dalbergioidin, 16-hydroxyhexadecanoic acid, and aromadendrin were purchased from Naturewill biotechnology Co., Ltd. (Chengdu, China). Niflumic acid, eriodictyol, and procyanidin B3 were purchased from Jiangsu Yongjian Pharmaceutical Bio-Technology Co., Ltd. (Taizhou, China). Taxifolin was purchased from Shanghai Yuanye Bio-Technology Co., Ltd. (Shanghai, China). All compounds with purity of higher than 98% were dissolved in dimethyl sulfoxide (DMSO, Sigma-Aldrich, St. Louis, MO, USA).

### 2.2. Preparation of Unfermented and Fermented Tea Extracts

Dried green tea leaves (120 g, Biluochun purchased from Heantang Trading Co., Ltd., Suzhou, China) were subjected to two consecutive extraction cycles. In each cycle, the leaves were infused with 600 mL of boiling distilled water for 15 min with a gentle stirring every 3 min. The infusions from both cycles were then combined and filtered to yield a green tea extract (GTE) with a final concentration of 10% (*w*/*v*, based on the initial mass of the tea leaves). Then, 0.5% and 1.0% GTE were prepared by diluting the 10% GTE with sterile water.

To investigate the effect of the initial concentration, separate parallel fermentations were set up for the 0.5% and 10% GTE solutions. Each was inoculated with *A. niger* RAF106 conidia (at the final concentration of 5 × 10^5^ conidia/mL) and incubated at 30 °C for 8 days with shaking at 220 rpm. Fermented samples were collected every two days, filtered, and stored at -20 °C alongside non-fermented controls.

### 2.3. Assay for C. elegans Lifespan Under Normal and Stressful Conditions

Eighty L1-stage worms were cultured on NGM agar plates supplemented with either OP50 (1.2 × 10^8^ CFU/mL) or OP50 co-incubated with tea extracts. The plates were maintained at 20 °C, and the worms were transferred daily to fresh plates with corresponding treatments during the progeny production phase, then every 2 days thereafter. To assess lifespan under thermal and oxidative stress, L1-stage worms were fed with OP50 or OP50-tea extract mixtures for 3 days, followed by exposure to either 37 °C or 2 mM H_2_O_2_ at 20 °C until death [10,27]. During cultivation, the worms were gently probed with a platinum wire pick under a stereoscopic microscope (Olympus SZX16, Tokyo, Japan), and individuals showing no response to three consecutive stimuli were recorded as dead. The number of live and dead worms in each group was used to calculate the mean lifespan using the formula reported by Wang et al. (2022) [28].

### 2.4. Analysis of Body Length and Body Bends of C. elegans

L1-stage worms were cultured on the NGM agar plates containing either OP50 or OP50-tea extract mixtures at 20 °C, as described above. Twenty randomly selected adult worms (3-day-old or 12-day-old) were subjected to body length and bends analyses. For body length, the worms were immobilized in 25 mM levamisole hydrochloride, then body length was recorded under an optical microscope (MOTIC, Xiamen, China). For body bends, the worms were washed with M9 buffer to remove bacteria, resuspended in M9 buffer, and allowed to swim freely in M9 buffer. After 30 s, the number of body bends per worm was counted within 20 s [10].

### 2.5. Assay for Lipid Accumulation, Mitochondrial Membrane Potential, and the Production of Reactive Oxygen Species (ROS), Glutathione (GSH), Malondialdehyde (MDA), Superoxide Dismutases (SODs) and Catalases (CATs)

Adult worms (3- or 16-day-old) were selected randomly to analyze lipid accumulation, mitochondrial membrane potential, and ROS production using Oil Red O staining, a JC-1 assay kit (Yuanye, Shanghai, China), and 2,7-dichlorodihydrofluorescein diacetate (H2-DCF-DA) staining, respectively, according to the procedure reported by Yang et al. (2024) [29].

The 3-day-old worms (*n* = 1000) were washed three times with M9 buffer, resuspended in ice-cold PBS buffer (pH 7.2), frozen in liquid nitrogen, and homogenized using a tissue grinder. The homogenate was centrifuged at 4 °C to collect the supernatants for subsequent analyses of the production of GSH, MDA, SODs, and CATs using a total glutathione assay kit (S0052), a lipid peroxidation MDA assay kit (S0131S), a total superoxide dismutase assay kit with WST-8 (S0101S), and a catalase assay kit (S0051), respectively, which were purchased from Beyotime (Shanghai, China), according to the manufacturer’s introduction.

### 2.6. Non-Targeted Metabolomic Analysis

Unfermented and fermented green tea extracts (six replicates per sample) were lyophilized using a vacuum freezing dryer (Scientz-10N/A, Ningbo, China). The solid samples (50 mg) were dissolved using 80% MeOH solution supplemented with 0.02 mg/mL of L-2-chlorophenylalanin, an internal standard, and ground, followed by low-temperature ultrasonic extraction for 30 min (5 °C, 40 kHz) and storage at −20 °C for 30 min. The samples were centrifuged at 4 °C to collect the supernatant, which was injected into a Thermo UHPLC-Q Exactive HF-X system (Thermo Fisher, Waltham, MA, USA) equipped with an ACQUITY HSS T3 column (100 mm × 2.1 mm i.d., 1.8 μm; Waters, Waltham, MA, USA) and eluted with 0.1% formic acid in water:acetonitrile (95:5, *v*/*v*) (solvent A) and 0.1% formic acid in acetonitrile:isopropanol:water (47.5:47.5, *v*/*v*) (solvent B) for LC-MS/MS analysis at Majorbio Bio-Pharm Technology Co. Ltd. (Shanghai, China) [22]. The equal volumes of all samples were mixed to prepare a pooled quality control sample (QC). The mass spectrometric data were collected using a Thermo UHPLC-Q Exactive HF-X Mass Spectrometer (Thermo Fisher, Waltham, MA, USA) equipped with an electrospray ionization (ESI) source operating in positive mode and negative mode. The temperature, sheath gas flow rate, aux gas flow rate, ion-spray voltage floating (ISVF), normalized collision energy, full MS resolution, MS/MS resolution, and a mass range were set at 425 °C, 50 arb, 13 arb, −3500 V in negative mode and 3500 V in positive mode, 20–40–60 V, 60,000, 7500, and 70–1050 *m*/*z*, respectively.

For metabolomics data analysis, Progenesis QI (Version 3.0, Waters Corporation, Milford, Waltham, MA, USA) software was used to perform the pretreatment of LC/MS raw data and export a three-dimensional data matrix in CSV format. The metabolites were identified by searching databases including HMDB (http://www.hmdb.ca/(accessed on 24 November 2022)), Metlin (https://metlin.scripps.edu/ (accessed on 24 November 2022)), and Majorbio Database, and then analyzed through the free online majorbio cloud platform (cloud.majorbio.com (accessed on 24 November 2022)). Principal component analysis (PCA) and orthogonal least partial squares discriminant analysis (PLS-DA) were performed using the R package “ropls” (Version 1.6.2). The metabolites with fold change (FC) > 2, variable importance in the projection (VIP) > 1, and *p* < 0.05 were determined as significantly different metabolites.

### 2.7. Statistical Analysis

All the experiments were conducted in triplicate. Replicate results were expressed as mean ± standard deviation (SD). The normality of the data was verified using the Shapiro–Wilk test, and equal variances were assessed with Levene’s test. Data analysis included one-factor analysis of variance, followed by Tukey’s HSD test when assumptions were met. Statistical significance was set at *p* < 0.05. If the data did not fit a normal distribution, a nonparametric Kruskal–Wallis test was used.

## 3. Results

### 3.1. Dynamic Changes in Anti-Aging Effects of Tea Extracts During the Liquid-State Fermentation of Green Tea Extracts (GTE) with A. niger RAF106

**Figure 1:**
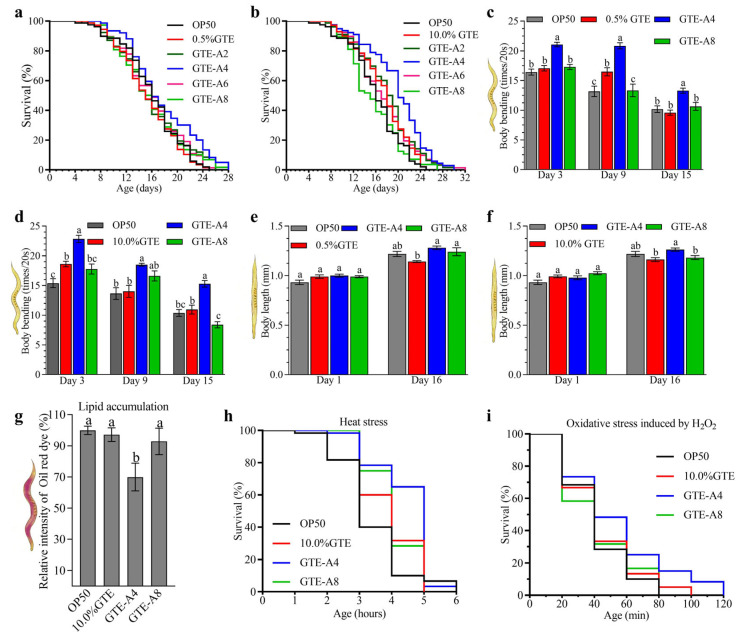
Effects of *A. niger* RAF106 fermentation of green tea extract (GTE) on the lifespan, body length, body bends, lipid accumulation, and stress resistance of *C. elegans* N2. (**a**–**f**) Changes in survival curves (**a**,**b**), body bends (**c**,**d**), and body length (**e**,**f**) when worms were fed with 0.5% (**a**,**c**,**e**) and 10.0% GTE (**b**,**d**,**f**) and their fermentation products mediated by RAF106 for 2 (GTE-A2), 4 (GTE-A4), 6 (GTE-A6), or 8 days (GTE-A8), respectively. (**g**–**i**) Effects of 10.0% GTE and its fermentation products on lipid accumulation (**g**) and survival of *C. elegans* N2 in response to 37 °C (**h**) and 2 mM H_2_O_2_ (**i**). Lipid accumulation was assessed by Oil Red O staining, and the fluorescence was quantified using ImageJ software (Version FIJI). Error bars: Standard deviation (SD) from replicates. Different lowercase letters on the bars of each group indicate significant differences among the group (*p* < 0.05).

In *C. elegans* N2, unfermented GTE at concentrations of 0.5–10.0% (*w*/*v*) failed to extend lifespan compared to the OP50 group (*p* > 0.05) (Figure 1a,b and Figure S1a). However, among GTE groups, 10.0% GTE significantly increased mean lifespan by 10.87% relative to 0.5% GTE (*p* < 0.05), whereas 1.0% GTE showed no effect (*p* > 0.05) (Figure S1a). In contrast, RAF106-fermented GTE extended lifespan in a dose- and fermentation time-dependent manner. Tea extracts from fermentation of 0.5% GTE by RAF106 for 4 days (0.5% GTE-A4) significantly increased mean lifespan by 11.62% (*p* < 0.05) compared to unfermented 0.5% GTE and by 8.61% (*p* < 0.05) compared to the OP50 group (Figure 1a and Figure S1b). Similarly, 10% GTE-A4 increased mean lifespan by 18.93% (*p* < 0.05) compared to the unfermented 10% GTE group and by 24.66% (*p* < 0.05) compared to the OP50 group (*p* < 0.05) (Figure 1b and Figure S1c). However, tea extracts from fermentation of GTE with RAF106 for 2, 6, and 8 days showed no significant effect on mean lifespan (*p* > 0.05), compared to the unfermented GTE group (Figure S1b,c), suggesting lifespan peaked at 4 days of fermentation and declined with longer fermentation. Given that lifespan promotion peaked at 4 days and declined thereafter, the 4-day (GTE-A4) and 8-day (GTE-A8) fermentation samples were selected for further assays to represent the peak of bioactivity and a subsequent time point of diminished effect.

Both 0.5% and 10% GTE significantly improved body bends by 20.94–25.00% only on day 9 and day 3 (*p* < 0.05), respectively, but did not affect body length (*p* > 0.05) in *C. elegans* N2 (Figure 1c–f) compared to the OP50 group. However, GTE-A4 significantly enhanced body bends and body length compared to unfermented GTE, while 0.5% GTE-A4 increased body bends by 23.45–38.65% over 15 days and body length by 12.02% in day-16 adults (*p* < 0.05) (Figure 1c,e). Similarly, 10% GTE-A4 increased body bends by 22.69–39.59% over the 15-day incubation and body length by 8.58% in day-16 adults (*p* < 0.05) (Figure 1d,f). However, 0.5% and 10% GTE-A8 impaired body bends (reductions of 14.29% on day 9 and 23.35% on day 15, respectively, *p* < 0.05) and had minimal or no effect on body length (Figure 1c–f). Notably, compared to the OP50 group, 0.5% and 10% GTE-A4 increased body bends by 28.32–67.41% and 35.53–48.38% (*p* < 0.05), respectively, while it showed no significant effect on body length (*p* > 0.05) (Figure 1c–f).

Based on its strong effects on lifespan and motility, 10% GTE and its fermented extracts were chosen for further assays. Compared to the OP50 group, unfermented GTE showed no significant effect on lipid accumulation (*p* > 0.05) but increased tolerance to 37 °C and H_2_O_2_ by 10.39% and 5.66% (*p* < 0.05), respectively, in *C. elegans* (Figure 1g–i and Figure S1d,e). In contrast, GTE-A4 lowered lipid accumulation by 30.07% and enhanced tolerance to 37 °C and H_2_O_2_ by 32.18% and 30.65% (*p* < 0.05), respectively, compared to the OP50 group (Figure 1g–i). However, GTE-A8 only exhibited an increase (19.58%) in tolerance to 37 °C (Figure 1g–i).

Collectively, RAF106 fermentation enhanced anti-aging properties of GTE in a concentration- and fermentation time-dependent manner, with 4-day fermented GTE-A4 showing the most pronounced benefits in *C. elegans* N2.

### 3.2. RAF106 Fermentation Modified the Impact of Tea Extracts on Mitochondrial Function and the Antioxidant Status of C. elegans

**Figure 2:**
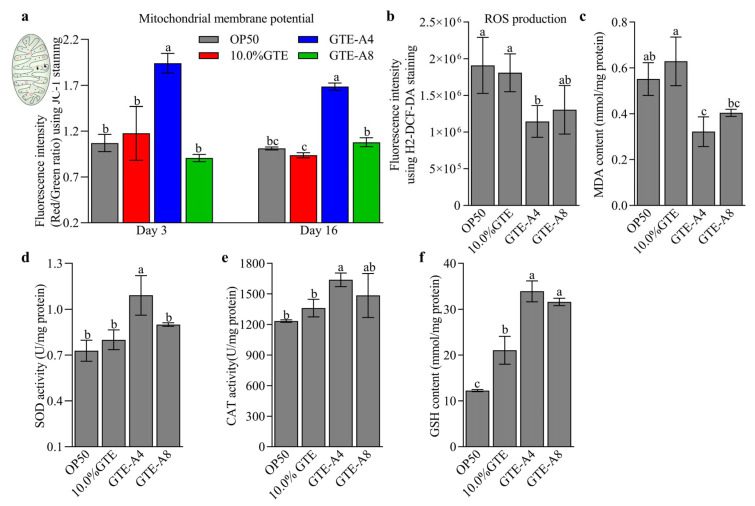
Effects of *A. niger* RAF106 fermentation on the regulatory effects of green tea extracts (GTE) regarding mitochondrial functions and antioxidant status in *C. elegans* N2. (**a**,**b**) Mitochondrial membrane potential assessed using JC-1 staining (**a**) and ROS production assessed using H2-DCF-DA staining (**b**) in worms fed with 10.0% GTE and its fermentation products mediated by *A. niger* RAF106 for 4 days (GTE-A4) or 8 days (GTE-A8). The fluorescence was quantified using ImageJ software (Version FIJI). (**c**–**f**) Effects of 10.0% GTE, GTE-A4, and GTE-A8 on MDA content (**c**), SOD activity (**d**), CAT activity (**e**), and GSH level (**f**). Error bars: Standard deviation (SD) from replicates. Different lowercase letters on the bars of each group indicate significant differences among the group (*p* < 0.05).

RAF106 fermentation could enhance the regulatory effects of green tea extracts on mitochondrial functions and antioxidant status in a fermentation time-dependent manner, with GTE-A4 exhibiting the most pronounced improvement in *C. elegans* N2. Compared to the OP50 group, unfermented GTE showed no significant effects on mitochondrial membrane potential (MMP, assessed by the ratio of the red-to-green fluorescence intensity in JC-1 staining), H2-DCF-DA fluorescence intensity (a marker of ROS), MDA level, and the activities of SODs and CATs (*p* > 0.05), but it increased GSH content by 71.84% (*p* < 0.05) (Figure 2a–f). In contrast, GTE-A4 significantly enhanced MMP by 65.09% and 80.02% in 3- and 16-day-old adults (*p* < 0.05), respectively, compared to unfermented GTE (Figure 2a). Concurrently, GTE-A4 reduced H2-DCF-DA fluorescence intensity and MDA level by 36.77% and 48.81%, respectively, and increased the production of SODs, CATs, and GSH by 36.37%, 32.86%, and 61.02%, respectively (*p* < 0.05) (Figure 2b–f). However, GTE-A8 only increased GSH content by 49.99% (*p* < 0.05) (Figure 2a–f). Notably, compared to the OP50 group, GTE-A4 exhibited significant increases in MMP (66.73–81.35%) and the production of GSH (176.70%), SODs (49.74%), and CATs (20.40%), along with significant reductions in ROS production (40.10%) and MDA level (41.64%) (*p* < 0.05) (Figure 2a–f).

### 3.3. RAF106-Fermented GTE (GTE-A4) Promoted Longevity via Multiple Pathways

**Figure 3:**
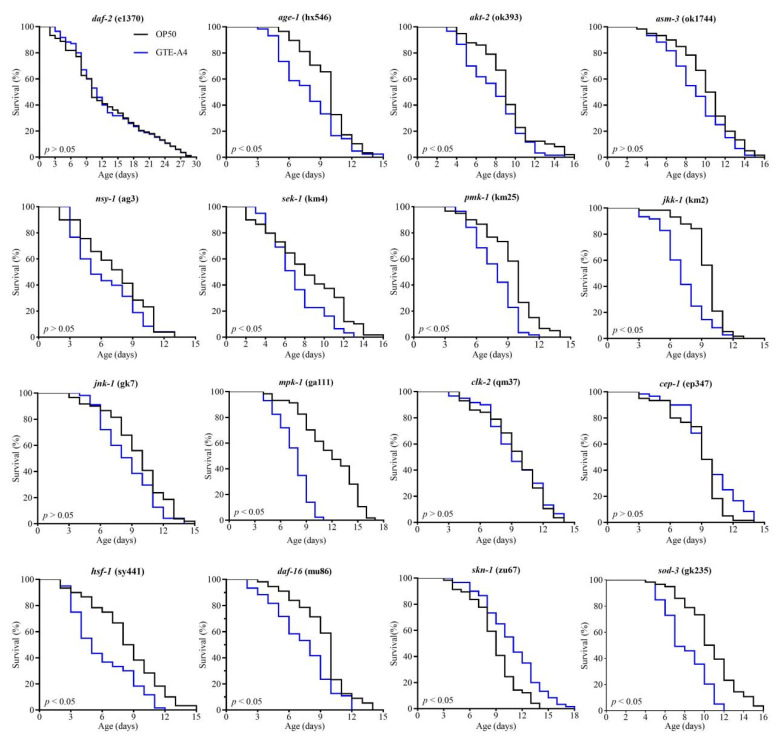
Effect of *A. niger* RAF106-fermented green tea extract (10% GTE, 4-day fermentation, GTE-A4) on the lifespan of various *C. elegans* longevity-pathway mutants. Loss-of-function mutants tested include: *daf-2*, *age-1*, *akt-2*, *asm-3*, *nsy-1*, *sek-1*, *pmk-1*, *jkk-1*, *jnk-1*, *mpk-1*, *clk-2*, *cep-1*, *hsf-1*, *daf-16*, *skn-1*, and *sod-3*.

As shown in Figure 3, administration with GTE-A4 failed to significantly extend the lifespan of worm mutants defective in *daf-2*, *age-1*, *asm-3*, *akt-2*, *nsy-1*, *sek-1*, *pmk-1*, *jkk-1*, *jnk-1*, *mpk-1*, *clk-2*, or *cep-1*, compared to the OP50 group. Similarly, no lifespan extension was observed in *hsf-1*, *daf-16*, or *sod-3* mutants. However, GTE-A4 significantly prolonged the mean lifespan of mutants defective in *skn-1* (*p* < 0.05). These results demonstrated GTE-A4 promoted longevity through IIS, p38 MAPK, ERK, JNK, and p53 pathways, with dependency on the transcription factors DAF-16 and HSF-1 (Figure S2).

### 3.4. RAF106 Fermentation Modified the Chemical Profile of GTE, Leading to the Enrichment of Numerous Longevity-Promoting Metabolites in GTE-A4

**Figure 4:**
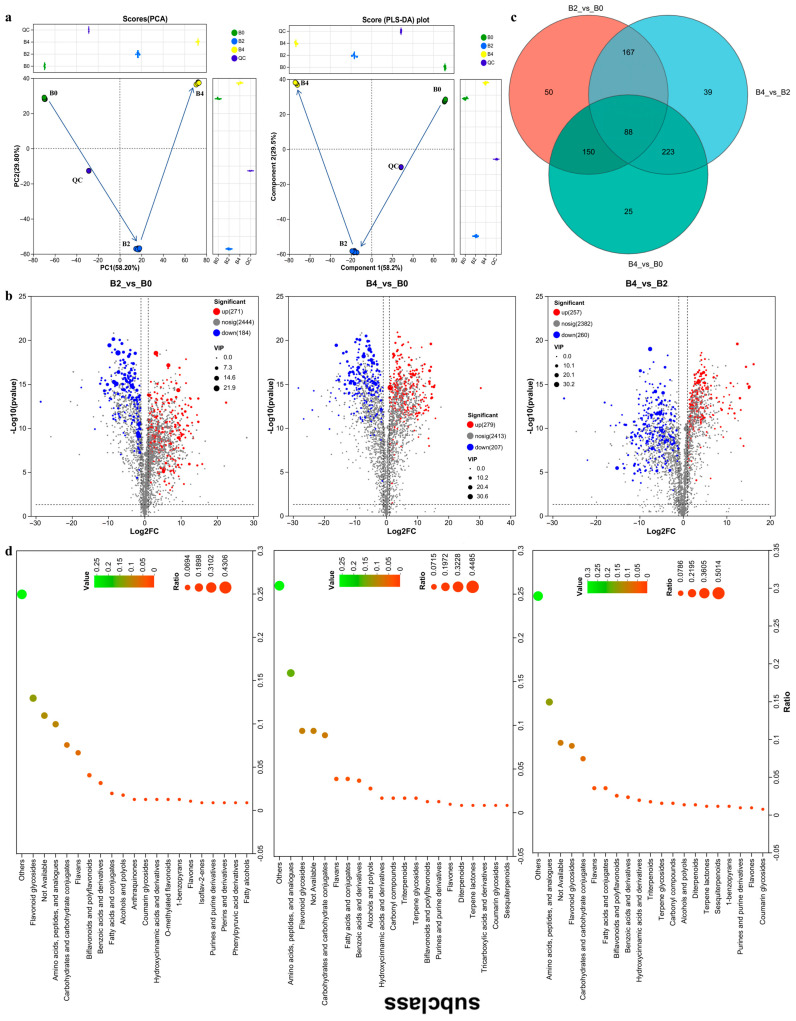
Analysis of metabolomics data from fermented green tea extracts. (**a**) Principal component analysis (PCA) and partial least squares-discriminant analysis (PLS-DA) score plots of the metabolome, which display distinct metabolic profiles among non-fermented (B0) and fermented GTE (B2/GTE-A4, B4/GTE-A8). The spatial arrangement of samples reflects their metabolic similarity, with closer points indicating more similar compositions. The arrows indicate the direction of metabolic change corresponding to increasing fermentation time. The adjacent boxes detail the distribution of samples along each component axis, reflecting the degree of clustering among samples. Each point represents an individual sample (*n* = 6). (**b**) Volcano plots of differential metabolites for the comparisons B2 vs B0, B4 vs B0, and B4 vs B2. (**c**) Venn diagram showing the unique and shared differential metabolites across the three comparisons. (**d**) HMDB subclass classification of the differential metabolites. Samples B0, B2, and B4 represented 10% GTE (B0), 10% GTE fermented by *A. niger* RAF106 for 4 days, and 10% GTE fermented for 8 days, respectively. QC: Quality Control.

To elucidate the differential anti-aging effects among GTE, GTE-A4, and GTE-A8, untargeted LC-MS/MS metabolomic profiling was conducted. A total of 2472, 2087, and 1973 metabolites were identified in GTE, GTE-A4, and GTE-A8, respectively (Table S1). Both PCA and PLS-DA score plots revealed clear differentiation among the three groups, indicating distinct metabolite profiles (Figure 4a). According to the threshold of FC > 2, VIP > 1, and *p* < 0.05, significant alterations in metabolite levels were observed following fermentation with *A. niger* RAF106. Compared to GTE, 455 (271 increased and 184 decreased) and 486 (279 increased and 207 decreased) metabolites were significantly altered in GTE-A4 and GTE-A8, respectively (Figure 4b). Comparison between GTE-A8 and GTE-A4 revealed significant alterations in 517 metabolites (257 increased and 260 decreased) (Figure 4b). Moreover, 88 core metabolites were common across all comparisons (Figure 4c). Annotated differential metabolites were predominantly categorized into flavonoid glycosides, amino acids/peptides and analogues, carbohydrates and carbohydrate conjugates, flavans, biflavonoids and polyflavonoids, benzoic acids and derivatives, fatty acids and conjugates, and alcohols and polyols (Figure 4d), according to HMDB databases.

**Figure 5:**
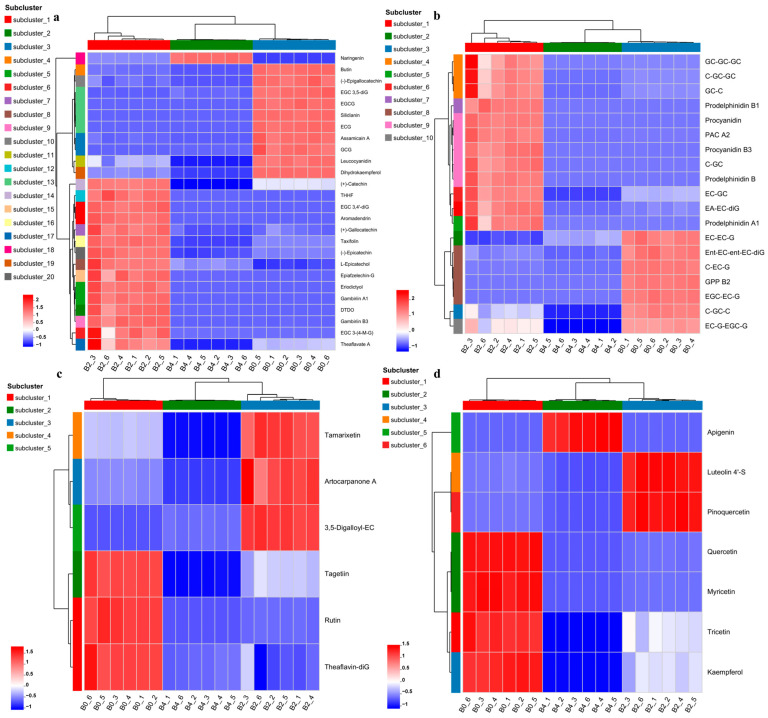
Heatmap analysis of metabolites belonging to flavans (**a**), bioflavonoids and polyflavonoids (**b**), O-methylated flavonoids (**c**), and flavones (**d**) in different samples. EGC 3,5-diG: Epigallocatechin 3,5-di-gallate. EGCG: Epigallocatechin gallate. ECG: Epicatechin 3-O-gallate. GCG: Gallocatechin 3-gallate. THHF: Trans-3,3′,4′,5,5′,7-Hexahydroxyflavanone. EGC 3,4′-diG: Epigallocatechin 3,4′-di-gallate. Epiafzelechin-G: Epiafzelechin 3-gallate. DTDO: (2R)-2-(3,4-Dihydroxyphenyl)-3,5,7-trihydroxy-2,3-dihydrochromen-4-one. EGC 3-(4-M-G): Epigallocatechin 3-(4-methyl-gallate). GC-GC-GC: Gallocatechin-(4alpha->8)-gallocatechin-(4alpha->8)-gallocatechin. C-GC-GC: Catechin-(4alpha->8)-gallocatechin-(4alpha->8)-gallocatechin. GC-C: [Gallocatechin(4alpha->8)]2catechin. PAC A2: Proanthocyanidin A2. C-GC: Catechin-(4->8)-gallocatechin. EC-GC: Epicatechin-(4beta->8)-gallocatechin. EA-EC-diG: Epiafzelechin-(4b->8)-epicatechin 3,3′-digallate. EC-EC-G: Epicatechin-(4beta->8)-epicatechin 3′-gallate. Ent-EC-ent-EC-diG: Ent-Epicatechin-(4alpha->8)-ent-epicatechin 3,3′-digallate. C-EC-G: Catechin-(4alpha->8)-epicatechin 3′-gallate. GPP B2: 3′-Galloylprodelphinidin B2. EGC-EC-G: Epigallocatechin-(4beta->8)-epicatechin 3-O-gallate. C-GC-C: Catechin-(4alpha->8)-gallocatechin-(4alpha->8)-catechin. EC-G-EGC-G: Epicatechin 3-O-gallate-(4beta->6)-epigallocatechin 3-O-gallate. 3,5-Digalloyl-EC: 3,5-Digalloylepicatechin. Theaflavin-diG: Theaflavin 3,3′-digallate. Luteolin 4′-S: Luteolin 4′-sulfate. Samples B0, B2, and B4 represented 10% GTE (B0), 10% GTE fermented by *A. niger* RAF106 for 4 days, and 10% GTE fermented for 8 days, respectively.

During fermentation, the composition of catechins in GTE underwent substantial biotransformation (Figure 5a). Galloylated catechins, such as EGCG, ECG, GCG, and epigallocatechin 3,5-di-gallate decreased markedly in GTE-A4. In contrast, their degalloylated counterparts, such as EC, GC, and C, increased temporarily in GTE-A4, followed by a decline in GTE-A8. EGC levels declined consistently throughout both fermentation stages. Additionally, a subset of other galloylated catechins (such as gallocatechin, epigallocatechin 3,4′-di-gallate, and epiafzelechin 3-gallate) increased transiently in GTE-A4 before eventually decreasing. Moreover, GTE-A4 accumulated significantly higher levels of aromadendrin, taxifolin, eriodictyol, and theaflavate A, while naringenin was most abundant in GTE-A8 (Figure 5a). Polymerized catechins present in GTE were degraded during fermentation, with new polymers forming by day 4 and decreasing by day 8 (Figure 5b). Several proanthocyanidins (such as prodelphinidin A1/B/B1, procyanidin B3, and proanthocyanidin A2) peaked in GTE-A4 (Figure 5b).

For O-methylated flavonoids, rutin, tagetiin, and theaflavin 3,3′-digallate decreased during fermentation, whereas tamarixetin, artocarpanone A, and 3,5-digalloylepicatechin reached maxima in GTE-A4 (Figure 5c). Additionally, 37 flavonoid glycosides (e.g., isoquercitrin, isoerocitrin, astragalin, quercetagitrin, astilbin, quercetin-3-glucoside) decreased following fermentation, while 30 other glycosides (e.g., vitexin, baohuoside I, vicenin 2, neoisoastilbin, quercetin 3-O-caffeyl-glucosede, peonidin-3-glucoside) peaked in GTE-A4 (Figure S3). Flavones including quercetin, myrivetin, tricetin, and kaempferol decreased (Figure 5d). Luteolin 4′-sulfate and pinoquercetin increased transiently in GTE-A4 before decreasing, while apigenin increased in GTE-A8 (Figure 5d).

Amino acids and peptides such as L-leucine, oxidized glutathione, L-glutamine, L-arginine, and L-glutamic acid decreased after fermentation (Figure S4). L-theanine, pantothenic acid, γ-glutamylproline increased in GTE-A4 but decreased in GTE-A8 (Figure S4). GTE-A8 showed elevated levels of 59 compounds, including L-coprine, kainic acid, aminoadipic acid, betaine, and Thr-Leu (Figure S4). In terms of carbohydrates and carbohydrate conjugates, 17 compounds (e.g., gentianose, lotaustralin, trehalose, validamycin A) decreased after fermentation, while 14 compounds (e.g., D-Apiose, benzoyl glucuronide, asperuloside, rutinose) increased in GTE-A4 and 21 compounds (e.g., acetic acid, rhamnose, muramic acid, arabinogalactose) increased in GTE-A8 (Figure S5).

Benzoic acids such as salicylic acid, methyl gallate, gallic acid, and vanillic acid decreased during fermentation (Figure S6a). Gentisic acid, 2,4,6-trihydroxybenzoic acid, 2,4-dihydroxybenzoic acid, and 2,3-dihydroxybenzoic acid increased transiently in GTE-A4 before declining, while terephthalic acid, 3-hydroxybenzoic acid, propyl gallate, isobutyl 4-hydroxybenzoate, heptyl 4-hydroxybenzoate, bergenin, and 2-pyrocatechuic acid increased in GTE-A8 (Figure S6a).

Purines and derivatives including adenine, xanthine, oxypurinol, caffeine, and theobromine increased progressively over 8 days, and guanine, 6-thiourate, 2-thiouric acid, 6-succinoaminopurine, and uric acids peaked in GTE-A4 (Figure S6b). In terms of hydroxycinamic acids and derivatives, carthamone and osmanthuside A decreased, while 4-coumaroylputrescine, coumarinic acid, and dattelic acid increased throughout fermentation (Figure S6c), and 1-O-sinapoyl-β-D-glucose, Mono-trans-p-coumaroylmesotartaric acid, and sinapic acid peaked in GTE-A4 (Figure S6c). In terms of fatty acids and conjugates, 16-hydroxyhexadecanoic acid and glycinoeclepin A peaked in GTE-A4, and 18 compounds, including pelargonic acid, tiglic acid, isovaleric acid, mevalonic acid, valeric acid, citramalic acid, monic acid, and traumatic acid increased in GTE-A8 (Figure S6d). Additionally, GTE-A4 contained the highest levels of niflumic acid, isovitexin, and dalbergioidin (Figure S6e).

**Figure 6:**
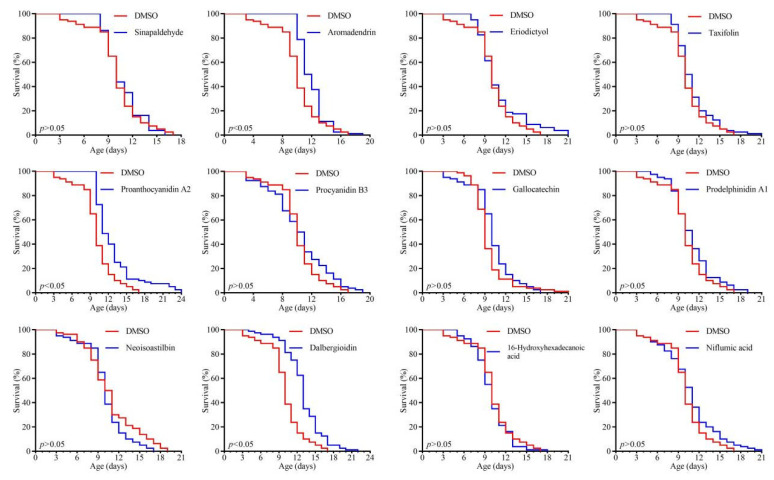
Effects of metabolites reaching peak concentrations during fermentation of 10% green tea extract by *A. niger* RAF106 for 4 days on lifespan in *C. elegans* N2. Key metabolites identified at peak levels included sinapaldehyde, aromadendrin, eriodictyol, taxifolin, proanthocyanidin A2, procyanidin B3, gallocatechin, prodelphinidin A1, neoisoastilbin, dalbergioidin, 16-hydroxyhexadecanoic acid, and niflumic acid. The final concentration of each compound was 10 mg/L.

Furthermore, to identify anti-aging compounds in RAF106-fermented GTE, compounds that peaked in GTE-A4 were selected for evaluation. Several compounds with known anti-aging effects, such as catechin, epicatechin, tamarixetin, vitexin, isovitexin, peonidin-3-glucoside, baohuoside I (Icariin II), L-theanine, and asperuloside, were included. Among other abundant compounds tested at 10 mg/L, proanthocyanidin A2, aromadendrin, and dalbergioidin significantly extended the mean lifespan of *C. elegans* N2 by 26.61%, 18.44%, and 28.59% (*p* < 0.05), respectively (Figure 6). In contrast, gallocatechin, prodelphinidin A1, neoisoastilbin, sinapaldehyde, eriodictyol, procyanidin B3, taxifolin, 16-hydroxyhexadecanoic acid, and niflumic acid had no significant lifespan extension (*p* > 0.05) compared to the DMSO control, which exhibited a mean lifespan comparable to the OP50 group.

Collectively, fermentation by *A. niger* RAF106 significantly altered the chemical composition of green tea extracts, leading to the enrichment of numerous longevity-promoting metabolites in GTE-A4, such as catechin, epicatechin, tamarixetin, vitexin, isovitexin, peonidin-3-glucoside, baohuoside I, L-theanine, asperuloside, proanthocyanidin A2, aromadendrin, and dalbergioidin.

## 4. Discussion

### 4.1. Fermentation with RAF106 for 4 Days Improved Anti-Aging Effects of GTE

As a globally consumed beverage, tea represents a promising source for developing nutrition-oriented anti-aging interventions [8]. Hot water extracts of green, white, yellow, oolong, black, and dark teas have been reported to extend lifespan, attenuate age-related mobility decline, reduce ROS and lipofuscin accumulation, and enhance stress tolerance in *C. elegans* [10]. Among the six types of tea, green tea exhibited superior effects [10]. However, the anti-aging effects of green tea varied by cultivar. Yiwu, Bulanshan, and Xigui cultivars extended lifespan, whereas Shuchazao and Hekai did not [10]. Here, hot water extract of Biluochun (GTE), a type of green tea, failed to extend mean lifespan in *C. elegans* N2 but increased body movement and tolerance to heat and H_2_O_2_-induced oxidative stress, which resembled the phenotypes reported for Shuchazao and Hekai teas [10]. The observed oxidative protection aligned with earlier findings that Biluochun extract alleviated Cr^6+^-induced oxidative stress in *C. elegans* [9]. These findings collectively indicate the limited anti-aging effects of unfermented Biluochun GTE under the present conditions.

Microbial fermentation can enhance the bioactivities of tea extracts or leaves. Previously, solid-state fermentation with *Eurotium cristatum* improved the hypoglycemic effect of Anji Baicha by inhibiting α-glucosidase and α-amylase [30]. Fermentation of white tea with *E. cristatum* and *Saccharomyces boulardii* enhanced its antioxidative capacity and improved skin health parameters clinically [31], while liquid-state green tea with *Ganoderma lucidum* enhanced lipid-lowering activity in mice [32]. Fermentation with *A. niger* was shown to increase the pancreatic lipase inhibitory activity of green tea extract [19]. Solid-state fermentation of Lu’an GuaPian (LGPT) with *C. militaris* for 10 days prolonged mean lifespan by 7.6% and improved UV tolerance in *C. elegans*, but shorter or longer fermentation times were ineffective [15]. In this study, liquid-state fermentation of Biluochun GTE with *A. niger* RAF106 for 4 days (GTE-A4) significantly extended mean lifespan and enhanced tolerance to heat shock and H_2_O_2_-induced oxidative stress in *C. elegans* compared to both the OP50 control and unfermented GTE. Similar to *C. militaris*-fermented LGPT, shorter or longer fermentation times did not improve these outcomes. The increased heat tolerance was similar to the phenomenon, but the increased H_2_O_2_ resistance differed from the unchanged paraquat-induced oxidative stress observed in *C. militaris*-fermented LGPT [15]. In addition to lifespan and stress tolerance, GTE-A4 increased body bends of *C. elegans* during a 16-day incubation, which was similar to the effects reported for other fermented teas but different from the unchanged body bends observed in *C. militaris*-fermented LGPT [10,15]. Moreover, body length increased in GTE-A4-fed worms compared to the GTE group, though not relative to OP50, aligning with reports from other fermented teas but differing from *C. militaris*-fermented LGPT [10,15].

The increase in longevity was accompanied by reduced lipid accumulation, a key age-related biomarker [32]. Reduction in fat accumulation induced by GTE-A4 aligned with previous studies showing that various tea extracts and *C. militaris*-fermented LGPT reduced fat content in *C. elegans* [10,15], and was consistent with the lipid-lowering effects of *G. lucidum*-fermented green tea in mice [33].

In summary, RAF106 fermentation for 4 days significantly enhanced the anti-aging efficacy of Biluochun GTE, improving both lifespan and healthspan in *C. elegans*.

### 4.2. RAF106-Fermented GTE-A4 Promoted Longevity via Multi-Pathways

Excessive ROS accumulation leads to oxidative stress, aging, and age-related diseases [34,35]. Feeding with GTE-A4 reduced ROS production and MDA level (a marker of oxidative damage) [36], consistent with the observed enhancement in tolerance to H_2_O_2_-induced stress and improvements in lifespan and healthspan. The elevated mitochondrial membrane potential in GTE-A4-fed worms further indicated improved mitochondrial function, which may underlie the reduction in ROS [37]. Furthermore, GTE-A4 boosted both enzymatic (SODs and CATs) and nonenzymatic (GSH) antioxidant defenses, key mechanisms that counterbalance oxidative stress [38].

In *C. elegans*, the IIS, p38 MAPK, JNK, and ERK pathways are associated with longevity [39,40]. GTE-A4 failed to extend lifespan in mutants defective in IIS (*daf-2*, *asm-3*, *age-1*, *akt-2*), p38 MAPK (*nsy-1*, *sek-1*, *pmk-1*), JNK (*jkk-1*, *jnk-1*), and ERK (*mpk-1*) pathways. The p38 MAPK and IIS pathways regulate the transcription factors SKN-1, DAF-16, and HSF-1 to modulate longevity and stress resistance [41,42,43,44,45]. JNK-1 and MPK-1/ERK directly interact with DAF-16, up-regulating downstream genes involved in metabolism, stress response, and aging [39,46,47,48]. Consistent with this, GTE-A4 did not extended lifespan in *daf-16* or *hsf-1* defective mutants, but remained effective in *skn-1* defective mutants. Moreover, the superoxide dismutase gene *sod-3*, a direct target of DAF-16, is essential for longevity [49]. The longevity-enhancing effects of GTE-A4 was abolished in *sod-3* mutants. Finally, the p53 homolog CEP-1 controls apoptosis and longevity, which might be regulated by CLK-2 in *C. elegans* [50,51]. GTE-A4 failed to extend lifespan in *clk-2* or *cep-1* defective mutants, implicating the p53 network in its mechanism.

These results indicated that GTE-A4 extends lifespan through improved mitochondrial function and reduced oxidative stress, operating via IIS, MAPK, and p53-related pathways in a manner dependent on DAF-16 and HSF-1.

### 4.3. Longevity-Promoting Metabolites in RAF106-Fermented GTE-A4

The health benefits of tea are largely attributed to its bioactive compounds, including catechins, flavonols, flavonoids, and flavonoid glycosides, anthocyanins, and phenolic acids [52,53]. Previously, RAF106 was shown to convert galloylated catechins (EGCG, ECG, GCG) into nongalloylated catechins (EGC, EC, GC) and GA, which could be degraded during long-term liquid-state fermentation [20,23]. Similarly, EGCG, ECG, GCG, EGC, GA, EGC-3,5-di-gallate, and some polymerized catechins in Biluochun GTE were degraded after fermentation with RAF106. Conversely, EC, GC, and C levels increased at 4 days of fermentation, but decreased by day 8. The GA degradation contrasted with the GA accumulation observed in *C. militaris-*fermented LGPT and liquid-state fermentation of instant dark tea by *E. cristatum*, and differed from the initial increase followed by a subsequent decrease in liquid-state fermentation of instant dark tea by *A. niger* [15,19,54]. Moreover, catechin monomers might be degraded into phenolic acids (e.g., 4-hydroxybenzoic acid, caffeic acid, and valeric acid) or undergo further polymerization during fermentation [19,54,55]. In this study, some polymerized catechins (e.g., proanthocyanidin A2, procyanidin B3, prodelphinidin B2, prodelphinidin A1, and prodelphinidin B), dihydroxybenzoic/trihydroxybenzoic acids, and theaflavate A peaked in GTE-A4. In contrast, 2-hydroxybenzoic acid, isovaleric acid, valeric acid, and mevalonic acid were accumulated after 8 days of fermentation. Flavonoid profiles were similarily altered. Rutin, quercetin, myricetin, and kaempferol decreased following RAF106 fermentation, while intermediates such as tamarixetin (quercetin 4′-O-methylether), pinoquercetin, aromadendrin, taxifolin, eriodictyol, and dalbergioidin peaked in GTE-A4, and apigenin and naringenin accumulated in GTE-A8. The decreases in rutin, quercetin, and myricetin aligned with observations in liquid-state fermentation of instant dark tea by *A. niger* [19]. However, the decrease in kaempferol differed from its transient increase (days 3 and 5) before decline in the same study. The accumulation of apigenin and naringenin observed here was opposite to their decrease reported by Sun et al. (2024) but similar to the increase in naringenin observed during liquid-state fermentation of instant dark teas in *A. niger* ZM1 [18,19]. Furthermore, previous studies indicated that during microbial fermentation of tea extracts or leaves, the glycosidic bond of flavonoid glycosides may be hydrolyzed, releasing sugar moieties and aglycones [15]. Conversely, microbial extracellular enzymes (e.g., cellulase, glucansucrase) can catalyze the glycosylation of flavonoids [56]. Consistent with these processes, this study revealed that 37 flavonoid glycosides decreased, while 30 other glycosides (e.g., vitexin, isovitexin, baohuoside I, penonidin-3-glucoside, neoisoastilbin) peaked in GTE-A4, and five glycosides accumulated in GTE-A8. These findings reflected the RAF106-induced modifications of flavonoids and flavonoid glycoside, including hydrolysis, glycosylation, deglycosylation, condensation, methylation, and dehydroxylation.

Among the differential compounds that peaked in GTE-A4, catechin, epicatechin, tamarixetin, vitexin, isovitexin, peonidin-3-glucoside, and baohuoside I have established roles in extending lifespan and stress resistance in *C. elegans* [57,58,59,60,61,62]. This study newly identified proanthocyanidin A2, aromadendrin, and dalbergioidin as longevity-promoting compounds. In contrast, gallocatechin, prodelphinidin A1, neoisoastilbin, sinapaldehyde, eriodictyol, procyanidin B3, and taxifolin failed to prolong lifespan. Additionally, L-theanine and asperuloside, which also peaked in GTE-A4, extended lifespan and delayed muscle aging, respectively, in *C. elegans* [63,64,65].

## 5. Conclusions

This study demonstrates that the unfermented GTE used herein failed to extend lifespan in *C. elegans*, but fermentation with *A. niger* RAF106 for 4 days (yielding GTE-A4) improved the anti-aging effects by enriching longevity-promoting metabolites, such as catechin, epicatechin, tamarixetin, vitexin, isovitexin, peonidin-3-glucoside, baohuoside I, L-theanine, asperuloside, proanthocyanidin A2, aromadendrin, and dalbergioidin. Compared to the OP50 or unfermented GTE, GTE-A4 significantly enhanced mean lifespan, body bends, stress resistance, mitochondrial function, and antioxidant properties, while reducing lipid accumulation and oxidative damage in *C. elegans*. The longevity benefits depended on insulin/IGF-1/p38 MAPK/JNK/ERK pathways, p53 network, and transcription factors DAF-16 and HSF-1. However, shorter or longer fermentation times diminished efficacy. Further studies should validate the anti-aging efficacy of GTE-A4 in mammalian models, identify additional longevity-promoting metabolites, and evaluate the effects of fermentation on flavor profiles.

## Data Availability

The original contributions presented in the study are included in the article/Supplementary Materials, further inquiries can be directed to the corresponding author.

## Supplementary Materials

The following supporting information can be downloaded at: https://www.mdpi.com/article/10.3390/foods14203548/s1, Figure S1. Effects of green tea extract (GTE) concentrations and *A. niger* RAF106-fermented GTE on lifespan in *C. elegans* N2. Figure S2. Proposed pathways underlying the beneficial effects induced by GTE-A4. Figure S3. Heatmap analysis of metabolites belonging to flavonoid glycosides in different samples. Figure S4. Heatmap analysis of metabolites belonging to amino acids, peptides, and analogue in different samples. Figure S5. Heatmap analysis of metabolites belonging to carbohydrates and carbohydrate conjugates in different samples. Figure S6. Heatmap analysis of metabolites belonging to benzoic acids and derivatives (a), purines and purine derivatives (b), hydroxycinamic acids and derivatives (c), fatty acids and conjugates (d), and relative content of dalbergloidin, eriodictyol, and niflumic acid (e) in different samples. Table S1. Metabolites overview.

## References

[1] Guo J., Huang X.Q., Dou L., Yan M.J., Shen T., Tang W.Q., Li J. (2022). Aging and aging-related diseases: From molecular mechanisms to interventions and treatments. Signal Transduct. Target. Ther..

[2] Yu Z.Y., Chen J., Yu R.F. (2024). Dose the increasing burden of social endowment affect sustainable development economy. PLoS ONE.

[3] Zhang J., Zhao Y., Sun Z., Sun T. (2022). *Lacticaseibacillus rhamnosus* Probio-M9 extends the lifespan of *Caenorhabditis elegans*. Community Biol..

[4] Xu Y., Song J., Huang Q., Wei X., Deng Z., Song Z., Huang H., Luo C., Zhang D., Han L. (2025). Functional foods and nutraceuticals with anti-aging effects: Focus on modifying the enteral microbiome. J. Funct. Foods.

[5] Bansal S., Choudhary S., Sharma M., Kumar S.S., Lohan S., Bhardwaj V., Syan N., Jyoti S. (2013). Tea: A native source of antimicrobial agents. Food Res. Int..

[6] Khan N., Mukhtar H. (2013). Tea and health: Studies in humans. Curr. Pharm. Des..

[7] Yan Z.M., Zhong Y.Z., Duan Y.H., Chen Q.H., Li F.N. (2020). Antioxidant mechanism of tea polyphenols and its impact on health benefits. Anim. Nutr..

[8] Xiang Y., Hu H., Chen H., Tang D., Huang Z., Zhang Y., Wang Z., Wang Z., Yangla, Han M. (2024). Tea consumption and attenuation of bilogical aging: A longitudinal analysis from two cohort studies. Lancet Reg. Health West Pac..

[9] Fei T., Fei J., Huang F., Xie T., Xu J., Zhou Y., Yang P. (2017). The anti-aging and anti-oxidation effects of tea water extract in *Caenorhabditis elegans*. Exp. Gerontol..

[10] Ke J., Li J., Yang Z., Wu H., Yu J., Yang Y., Chen C., Zhou P., Hua F., Wang W. (2024). Unraveling anti-aging mystery of green tea in *C. elegans*: Chemical truth and multiple mechanisms. Food Chem..

[11] Sarıtaş S., Portocarrero A.C.M., López J.M.M., Lombardo M., Koch W., Raposo A., EI-Seedi H.R., de Brito Alves J.L., Esatbeyoglu T., Karav S. (2024). The impact of fermentation on the antioxidant activity of food products. Molecules.

[12] Zhao D., Shah N.P. (2016). Lactic acid bacterial fermentation modified phenolic composition in tea extracts and enhanced their antioxidant activity and cellular uptake of phenolic compounds following in vitro digestion. J. Funct. Food..

[13] Kim M.J., John K.M.M., Choi J.N., Lee S., Kim A.J., Kim Y.M., Lee C.H. (2013). Changes in secondary metabolites of green tea during fermentation by *Aspergillus oryzae* and its effect on antioxidant potential. Food Res. Int..

[14] Kritsadaruangchai U., Chaiwut P., Chomnunti P., Thaochan N., Saikeur A., Pintathong P. (2019). Effect of solid state fermentation with *Trichoderma* spp. on phenolic content and antioxidant capacities of mature Assam tea leaves. J. Food Sci. Agric. Technol..

[15] Ke J., Zhang Y., Li J., Wu H., Yu J., Chen C., Yang Y., Wang W., Hu F., Bao G. (2024). *Cordyceps militaris* fermentation changes the flavor and chemical profiles of Lu’an GuaPian green tea with fat-lowering and anti-aging activities. Microchem. J..

[16] Cai M., Huang L., Dong S., Diao N., Ye W., Peng Z., Fang X. (2023). Enhancing the flavor profile of summer green tea via fermentation with *Aspergillus niger* RAF106. Foods.

[17] Chen S., Fu Y., Bian X., Zhao M., Zuo Y., Ge Y., Xiao Y., Xiao J., Li N., Wu J. (2022). Investigation and dynamic profiling of oligopeptides, free amino acids and derivatives during Pu-erh tea fermentation by ultra-high performance liquid chromatography tandem mass spectrometry. Food Chem..

[18] Liao S., Zhao Y., Jia W., Niu L., Bouphun T., Li P., Chen S., Chen W., Tang D., Zhao Y. (2023). Untargeted metabolomics and quantification analysis reveal the shift of chemical constituents between instant dark teas individually liquid-state fermented by *Aspergillus cristatus*, *Aspergillus niger*, and *Aspergillus tubingensis*. Front. Microbiol..

[19] Sun H., Fan R., Fang R., Shen S., Wang Y., Fu J., Hou R., Sun R., Bao S., Chen Q. (2024). Dynamics changes in metabolites and pancreatic lipase inhibitory ability of instant dark tea during liquid-state fermentation by *Aspergillus niger*. Food Chem..

[20] Fang X., Du M., Liu T., Fang Q., Liao Z., Zhong Q., Chen J., Meng X., Zhou S., Wang J. (2019). Changes in the biotransformation of green tea catechins induced by different carbon and nitrogen sources in *Aspergillus niger* RAF106. Front. Microbiol..

[21] Fang Q., Du M., Chen J., Liu T., Zheng Y., Liao Z., Zhong Q., Wang L., Fang X., Wang J. (2020). Degradation and detoxification of aflatoxin B1 by tea-derived *Aspergillus niger* RAF106. Toxins.

[22] Liu T., Wang J., Du M., Wang Y., Fang X., Peng H., Shi Q., Xie X., Zhou G. (2022). The interplays between epigallocatechin-3-gallate (EGCG) and *Aspergillus niger* RAF106 based on metabolism. Fungal Biol..

[23] Liu T., Zhou G., Du M., Zhang X., Zhou S., Chen G., Liao Z., Zhong Q., Wang L., Xu X. (2023). The interplay between (-)-epigallocatechin-3-gallate (EGCG) and *Aspergillus niger* RAF106, an EGCG-biotransforming fungus derived from Pu-erh tea. LWT-Food Sci. Technol..

[24] Cai M., Peng Z., Xu P., Yu M., Diao N., Cao Y., Dong S., Fang X. (2025). Comprehensive analysis of the flavor and color characteristics of light-fermented sour tea mediated by *Aspergillus niger* RAF106. Food Chem..

[25] Wang Y., Zhang M., Zhang Z., Jiang J., Gao X., Yue P. (2018). Multiple responses optimization of instant dark tea production by submerged fermentation using response surface methodology. J. Food Sci. Technol..

[26] Kirchweger B., Zwirchmayr J., Grienke U., Rollinger J.M. (2023). The role of *Caenorhabditis elegans* in the discovery of natural products for healthy aging. Nat. Prod. Rep..

[27] Stenvall J., Fierro-Gonzalez J.C., Swoboda P., Saamarthy K., Cheng Q., Cacho-Valadez B., Arnér E.S.J., Persson O.P., Miranda-Vizuete A., Tuck S. (2011). Selenoprotein TRXR-1 and GSR-1 are essential for removal of old cuticle during molting in *Caenorhabditis elegans*. Proc. Natl. Acad. Sci. USA.

[28] Wang W.Q., Li S.P., Heng X., Chu W.H. (2022). *Weissella confusa* CGMCC 19,308 Strain protects against oxidative stress, increases lifespan, and bacterial disease resistance in *Caenorhabditis elegans*. Probiotics Antimicrob. Proteins.

[29] Yang X., Chen J., Liao Z., Xia Z., Huang H., Huang J., Chen L., Fang X., Gao C., Wang J. (2024). *Lactobacillus fermentum* WC2020 increased the longevity of *Caenorhabditis elegans* via JNK-mediated antioxidant pathway. J. Food Sci..

[30] Qiu Y., Liu X., Huang Z., Lyu F., Hu X., Han S., Ren H., Zhang A. (2025). Effect of *Eurotium cristatum* fermentation on chemincal composition and hypoglycemic and sedative activities of Anji Baicha (*Camellia sinensis*). J. Food Sci..

[31] Wang G., Fei W., Zhi L., Bai X., You B. (2024). Fermented tea leave extract against oxidative stress and ageing of skin in vitro and in vivo. Int. J. Cosmet. Sci..

[32] Johnson A.A., Stolzing A. (2019). The role of lipid metabolism in aging, lifespan regulation, and age-related disease. Aging Cell.

[33] Liu X., Ju Y., Zeng H., Wen S., Wang C., Jiang M., Tian B., Huang J., Liu Z. (2025). Green tea fermented by *Ganoderma lucidum* presented anti-obesity properties via enhanced thermogenesis in vitro and on C57BL/6J mice. Food Res. Int..

[34] Li W., Gao L., Huang W., Ma Y., Muhammad I., Hanif A., Ding Z., Guo X. (2022). Antioxidant properties of lactic acid bacteria isolated from traditional fermented yak milk and their probiotic effects on the oxidative senescence of *Caenorhabditis elegans*. Food Funct..

[35] Liguori I., Russo G., Curcio F., Bulli G., Aran L., Della-Morte D., Gargiulo G., Testa G., Cacciatore F., Bonaduce D. (2018). Oxidative stress, aging, and diseases. Clin. Interv. Aging.

[36] Gil L., Siems W., Mazurek B., Gross J., Schroeder P., Voss P., Grune T. (2006). Age-associated analysis of oxidative stress parameters in human plasma and erythrocytes. Free Radic. Res..

[37] Kumar A., Joishy T., Das S., Kalita M.C., Mukherjee A.K., Khan M.R. (2022). A potential probiotic *Lactobacillus plantarum* JBC5 improves longevity and healthy aging by modulating antioxidative, innate immunity and serotonin-signaling pathways in *Caenorhabditis elegans*. Antioxidants.

[38] Nakagawa H., Shiozaki T., Kobatake E., Hosoya T., Moriya T., Sakai F., Taru H., Miyazaki T. (2015). Effects and mechanisms of prolongevity induced by *Lactobacillus gasseri* SBT2055 in *Caenorhabditis elegans*. Aging Cell.

[39] Poupet C., Chassard C., Nivoliez A., Bornes S. (2020). *Caenorhabditis elegans*, a host to investigate the probiotic properties of beneficial microorganisms. Front. Nutr..

[40] Yoon D.S., Cha D.S., Choi Y., Lee J.W., Lee M.H. (2019). MPK-1/ERK is required for the full activity of resveratrol in extended lifespan and reproduction. Aging Cell.

[41] Chiang W., Ching T., Lee H.C., Mousigian C., Hsu A. (2012). HSF-1 regulators DDL-1/2 link insulin-like signaling to heat-shock responses and modulation of longevity. Cell.

[42] Kaletsky R., Lakhina V., Arey R., Williams A., Landis J., Ashraf J., Murphy C.T. (2016). The *C. elegans* adult neuronal IIS/FOXO transcriptome reveals adult phenotype regulators. Nature.

[43] Keshet A., Mertenskötter A., Winter S.A., Brinkmann V., Dölling R., Paul R.J. (2017). PMK-1 p38 MAPK promotes cadmium stress resistance, the expression of SKN-1/Nrf and DAF-16 target genes, and protein biosynthesis in *Caenorhabditis elegans*. Mol. Genet. Genom..

[44] Mertenskötter A., Keshet A., Gerke P., Paul R.J. (2013). The p38 MAPK PMK-1 shows heat-induced nuclear translocation, supports chaperone expression, and affects the heat tolerance of *Caenorhabditis elegans*. Cell Stress Chaperones.

[45] Tullet J.M.A., Hertweck M., An J.H., Baker J., Hwang J.Y., Liu S., Oliveira R.P., Baumeister R., Blackwell T.K. (2008). Direct inhibition of the longevity-promoting factor SKN-1 by Insulin-like signaling in *C*. elegans. Cell.

[46] Bianco J.N., Schumacher B. (2018). MPK-1/ERK pathway regulates DNA damage response during development through DAF-16/FOXO. Nucleic Acids Res..

[47] Oh S.W., Mukhopadhyay A., Svrzikapa N., Jiang F., Davis R.J., Tissenbaum H.A. (2005). JNK regulates lifespan in *Caenorhabditis elegans* by modulating nuclear translocation of forkhead transcription factor/DAF-16. Proc. Natl. Acad. Sci. USA.

[48] Tambara A.L., de Los S.M.L., Dal Forno A.H. (2018). Purple pitanga fruit (*Eugenia uniflora*, L) protects against oxidative stress and increase the lifespan in *Caenorhabditis elegans* via the DAF-16/FOXO pathway. Food Chem. Toxicol..

[49] Li N., Li X., Shi Y., Gao J., He Y., Li F., Shi J., Gong Q. (2021). Trilobatin, a component from Lithocarpus polystachyrus Rehd., increased longevity in C. elegans through activating SKN1/SIRT3/DAF16 signaling pathway. Front. Pharmacol..

[50] Arum O., Johnson T.E. (2007). Reduced expression of the *Caenorhabditis elegans* p53 ortholog *cep-1* results in increased longevity. J. Gerontol. A Biol. Sci. Med. Sci..

[51] Derry W.B., Bierings R., van Iersel M., Satkunendran T., Reinke V., Rothman J.H. (2007). Regulation of developmental rate and germ cell proliferation in *Caenorhabditis elegans* by the p53 gene network. Cell Death Differ..

[52] Liu Y., Zhao G., Li X., Shen Q., Wu Q., Zhuang J., Zhang X., Xia E., Zhang Z., Qian Y. (2020). Comparative analysis of phenolic compound metabolism among tea plants in the section Thea of the genus *Camellia*. Food Res. Int..

[53] Sanlier N., Gokcen B.B., Altuğ M. (2018). Tea consumption and disease correlations. Trends Food Sci. Technol..

[54] An T., Chen M., Zu Z., Chen Q., Lu H., Yue P., Gao X. (2021). Untargeted and targeted metabolomics reveal changes in the chemical constituents of instant dark tea during liquid-state fermentation by *Eurotium cristatum*. Food Res. Int..

[55] Takagaki A., Nanjo F. (2010). Metabolism of (-)-epigallocatechin gallate by rat intestinal flora. J. Agric. Food Chem..

[56] Zhu M., Li N., Zhou F., Ouyang J., Lu D., Xu W., Li J., Lin H., Zhang Z., Xiao J. (2020). Microbial bioconversion of the chemical components in dark tea. Food Chem..

[57] Cai W., Huang J., Zhang S., Wu B., Kapahi P., Zhang X., Shen Z. (2011). Icariin and its derivative icariside II extend healthspan via Insulin/IGF-1 pathway in *C. elegans*. PLoS ONE.

[58] Malar D.S., Prasanth M.I., Jeyakumar M., Balamurugan K., Devi K.P. (2020). Vitexin prevents Aβ proteotoxicity in transgenic *Caenorhabditis elegans* model of Alzheimer’s disease by modulating unfolded protein response. J. Biochem. Mol. Toxicol..

[59] Nas J.S.B., Manalo R.V.M., Medina P.M.B. (2021). Peonidin-3-glucoside extends the lifespan of *Caenorhabditis elegans* and enhances its tolerance to heat, UV, and oxidative stresses. ScienceAsia.

[60] Okoro N.O., Odiba A.S., Osadebe P.O., Omeje E.O., Liao G., Fang W., Jin C., Wang B. (2021). Bioactive phytochemicals with anti-aging and lifespan Extending potentials in *Caenorhabditis elegans*. Molecules.

[61] Surco-Laos F., Cabello J., Gómez-Orte E., González-Manzano S., González-Paramás A.M., Santos-Buelga C., Dueñas M. (2011). Effects of *O*-methylated metabolites of quercetin on oxidative stress, thermotolerance, lifespan and bioavailability on *Caenorhabditis elegans*. Food Funct..

[62] Tao M., Li R., Xu T., Zhang Z., Wu T., Pan S., Xu X. (2021). Flavonoids from the mung bean coat promote longevity and fitness in *Caenorhabditis elegans*. Food Funct..

[63] Chen Q., Zhang Q., Amrouche A.T., Huang W., Lu B. (2023). Asperuloside, the bioactive compound in the edible *Eucommia ulmoides* male flower, delays muscle aging by *daf-16* mediated improvement in mitochondrial dysfunction. Food Funct..

[64] Chen L., Chen G., Gai T., Zhou X., Zhu J., Wang R., Wang X., Guo Y., Wang Y., Xie Z. (2024). L-Theanine prolongs the lifespan by activating multiple molecular pathways in ultraviolet C-exposed *Caenorhabditis elegans*. Molecules.

[65] Zarse K., Jabin S., Ristow M. (2012). L-Theanine extends lifespan of adult *Caenorhabditis elegans*. Eur. J. Nutr..

