# Supplementary material for: Enhancing the Anti-Aging Potential of Green Tea Extracts Through Liquid-State Fermentation with Aspergillus niger RAF106
Source: Foods. 2025 Oct 18;14(20):3548. doi: 10.3390/foods14203548 (PMC12562963; doi:10.3390/foods14203548)
Supplement: Supplementary file 1 [file foods-14-03548-s001.zip › foods-3922348-supplementary.pdf]

**a**

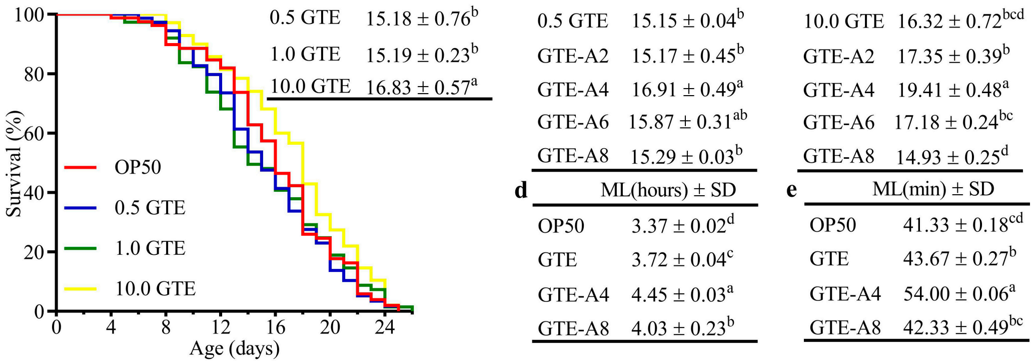


| | ML(days) ± SD |
|---|---|
| OP50 | $15.75 \pm 0.31^{ab}$ |
| 0.5 GTE | $15.18 \pm 0.76^{b}$ |
| 1.0 GTE | $15.19 \pm 0.23^{b}$ |
| 10.0 GTE | $16.83 \pm 0.57^{a}$ |

**b**

| | ML(days) ± SD |
|---|---|
| OP50 | $15.57 \pm 0.25^{b}$ |
| 0.5 GTE | $15.15 \pm 0.04^{b}$ |
| GTE-A2 | $15.17 \pm 0.45^{b}$ |
| GTE-A4 | $16.91 \pm 0.49^{a}$ |
| GTE-A6 | $15.87 \pm 0.31^{ab}$ |
| GTE-A8 | $15.29 \pm 0.03^{b}$ |

**c**

| | ML(days) ± SD |
|---|---|
| OP50 | $15.57 \pm 0.25^{cd}$ |
| 10.0 GTE | $16.32 \pm 0.72^{bcd}$ |
| GTE-A2 | $17.35 \pm 0.39^{b}$ |
| GTE-A4 | $19.41 \pm 0.48^{a}$ |
| GTE-A6 | $17.18 \pm 0.24^{bc}$ |
| GTE-A8 | $14.93 \pm 0.25^{d}$ |

**d**

| | ML(hours) ± SD |
|---|---|
| OP50 | $3.37 \pm 0.02^{d}$ |
| GTE | $3.72 \pm 0.04^{c}$ |
| GTE-A4 | $4.45 \pm 0.03^{a}$ |
| GTE-A8 | $4.03 \pm 0.23^{b}$ |

**e**

| | ML(min) ± SD |
|---|---|
| OP50 | $41.33 \pm 0.18^{cd}$ |
| GTE | $43.67 \pm 0.27^{b}$ |
| GTE-A4 | $54.00 \pm 0.06^{a}$ |
| GTE-A8 | $42.33 \pm 0.49^{bc}$ |

**Fig. S1.** Effects of green tea extract (GTE) concentrations and *A. niger* RAF106-fermented GTE on lifespan in *C. elegans* N2. (a) Survival curve and mean lifespan of worms fed with 0.5%, 1.0%, and 10.0% (w/v) GTE. (b, c) Mean lifespan of worms fed with 0.5% GTE (b) or 10.0% GTE (c) and their fermentation products (GTE-A2, GTE-A4, GTE-A6, and GTE-A8), compared to OP50. (d, e) Effects of 10.0% GTE and its fermentation products on mean lifespan under heat stress (37°C) (d) and oxidative stress (2 mM $H_2O_2$) (e). Error bars: Standard deviation (SD) from replicates. Different lowercase letters on the bars of each group indicate significant differences among the group ($p < 0.05$).

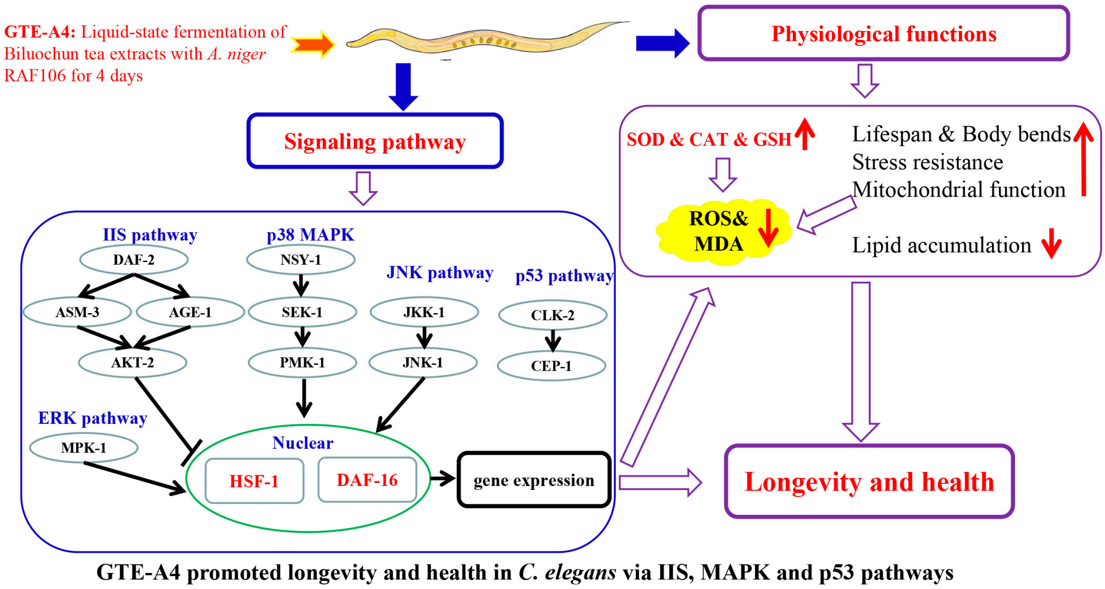


**Fig. S2.** Proposed pathways underlying the benefical effects induced by GTE-A4.

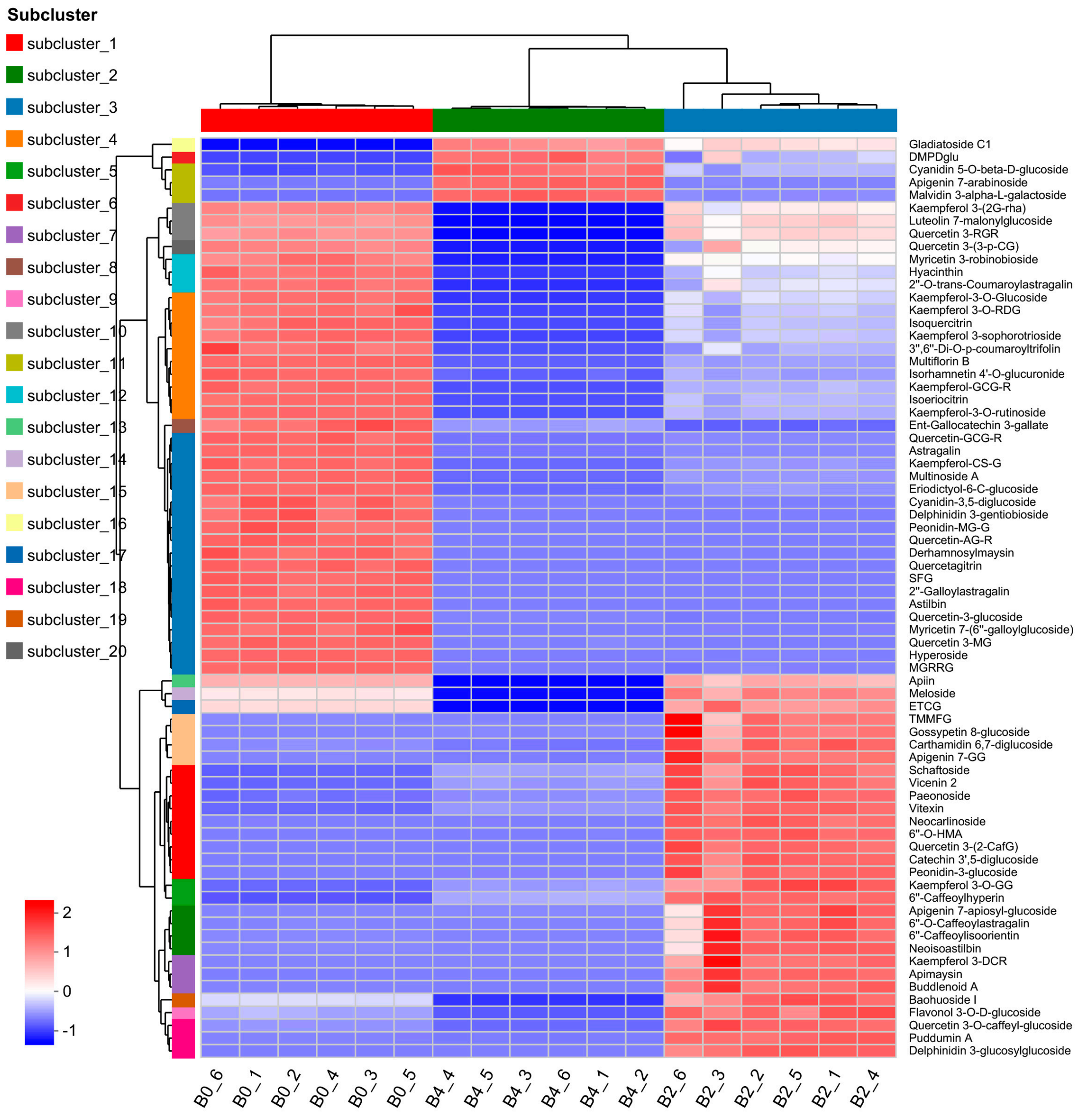


**Fig. S3.** Heatmap analysis of metabolites belonging to flavonoid glycosides in different samples. DMPDglu: 2',7-Dihydroxy-4'-methoxy-8-prenylflavan 2',7-diglucoside. Kaempferol 3-(2G-rha): Kaempferol 3-(2G-rhamnosylgentiobioside). Quercetin-GCG-R: Quercetin 3-(6-[4-glucosyl-p-coumaryl]glucosyl)(1->2)-rhamnoside. Quercetin 3-(3-p-CG): Quercetin 3-(3-p-coumaroylglucoside). Kaempferol 3-O-RDG: Kaempferol 3-O-rhamnodiglucoside. Kaempferol-GCG-R: Kaempferol

3-(6-[4-glucosyl-p-coumaryl]glucosyl)(1->2)-rhamnoside. Kaempferol-CS-G: Kaempferol 3-(2-p-coumaroylsophoroside) 7-glucoside. Peonidin-MG-G: Peonidin 3-(6''-malonyl-glucoside) 5-glucoside. Quercetin-AG-R: Quercetin 3-(6''-acetyl-galactoside) 7-rhamnoside. SFG: Spinacetin 3-(2''-feruloylgentiobioside). Quercetin 3-MG: Quercetin 3-(6''-malonyl-glucoside). MGRRG: Myricetin 3-[glucosyl-(1->2)-rhamnoside] 7-[rhamnosyl-(1->2)-glucoside]. ETCG: Eriodictyol 7-(6-trans-p-coumaroylglucoside). TMMFG: 3,3',5-Trihydroxy-4'-methoxy-6,7-methylenedioxyflavone 3-glucuronide. Apigenin 7-GG: Apigenin 7-glucuronosyl-glucoside. 6''-O-HMA: 6''-O-(3-Hydroxy-3-methylglutaroyl)astragalin. Quercetin 3-(2-CafG): Quercetin 3-(2-caffeoylglucuronoside). Kaempferol 3-O-GG: Kaempferol 3-O-beta-D-glucosyl-(1->2)-glucoside. Kaempferol 3-DCR: Kaempferol 3-(3'',6''-diacetyl-2'',4''-di-p-coumaroylrhamnoside). Samples B0, B2, and B4 represented 10% GTE (B0) , 10% GTE fermented by *A. niger* RAF106 for 4 days, and 10% GTE fermented for 8 days.

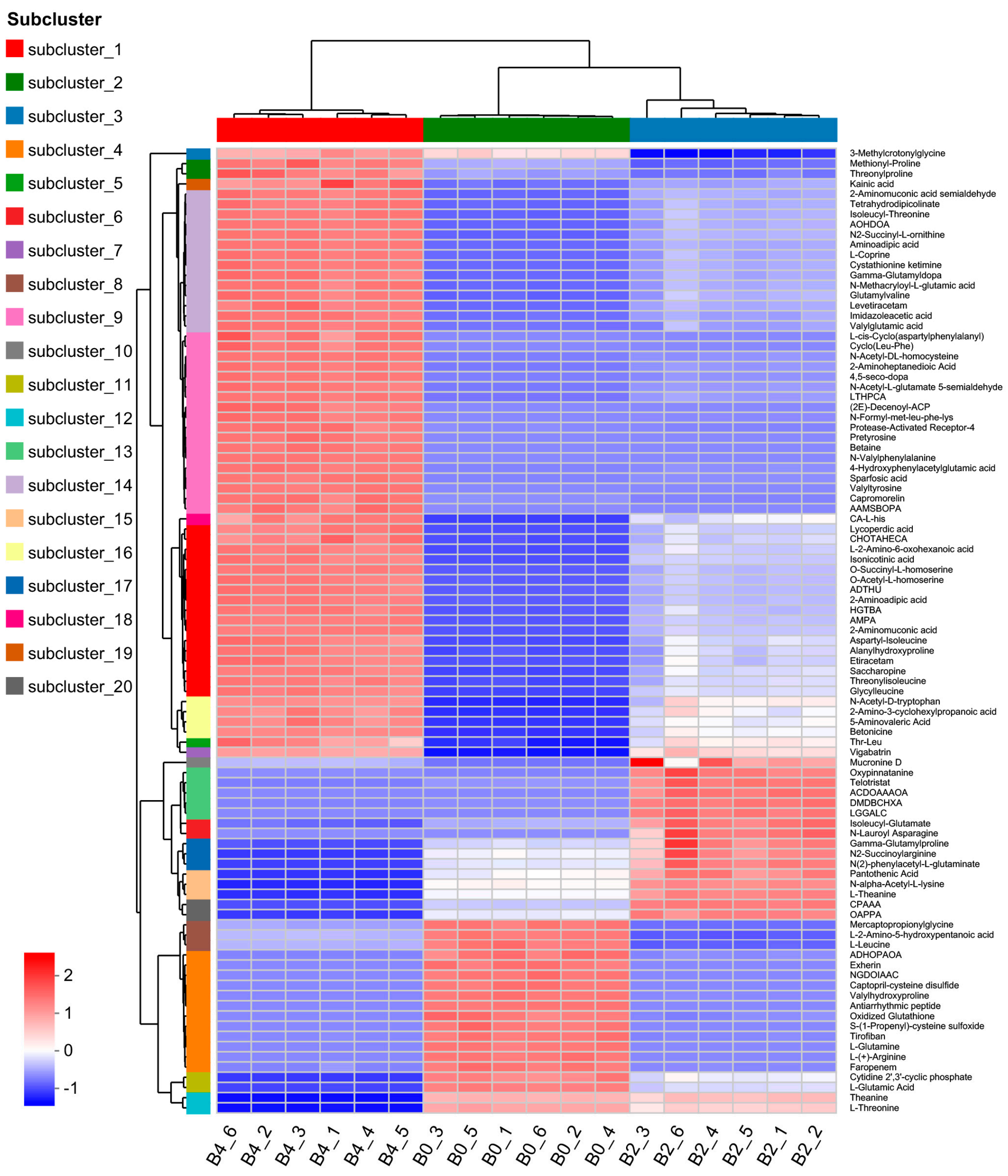


**Fig. S4.** Heatmap analysis of metabolites belonging to amino acids, peptides, and analogue in different samples. AOHDOA: (1R,4R,5S,6R)-4-Amino-2-oxabicyclohexane-4,6-dicarboxylic acid. LTHPCA: L-trans-5-Hydroxy-2-piperidinecarboxylic acid. AAMSBOPA: (2S)-2-Amino-3-[4-[(2S)-2-amino-4-methylsulfanylbutanoyl]oxyphenyl]propanoic. CA-L-his: 2-(3-Carboxy-3-aminopropyl)-L-histidine. CHOTAHECA: (5R,6S)-3-(2-Carbamoyloxyethylsulfanyl)-6-(1-hydroxyethyl)-7-oxo-4-thia-1-azabicy

clohept-2-ene-2-carboxylic acid. ADTHU: 3-amino-3,7-dideoxy-D-threo-hept-6-ulosonate. HGTBA: Beta-Hydroxy-gamma-trimethylaminobutyric acid. AMPA: L-2-Amino-4-methylenepentanedioic acid. ACDOAAAOA: 3-Amino-5-[[2-[[1-chloro-6-(diaminomethylideneamino)-2-oxohexan-3-yl]amino]acetyl]amino]-5-oxopentanoic acid. DMDBCHXA: (2S)-2-[[4-[(2,4-Diaminopteridin-6-yl)methyl]-2,3-dihydro-1,4-benzothiazine-7-carbonyl]amino]hexanedioic acid. LGGALC: L-gamma-Glutamyl-S-allylthio-L-cysteine. CPAAA: 2-[Carbamimidoyl(phosphonomethyl)amino]acetic acid. OAPPA: (R)-2-Amino-3-(2-propynylthio)propanoic acid. ADHOPAOA: 4-[2-[2-[3-Acetamido-2,5-dihydroxy-6-(hydroxymethyl)oxan-4-yl]oxypropanoylamino]propanoylamino]-5-amino-5-oxopentanoic acid. NGDOIAAC: N-[[3-(b-D-Glucopyranosyloxy)-2,3-dihydro-2-oxo-1H-indol-3-yl]acetyl]aspartic acid. Samples B0, B2, and B4 represented 10% GTE (B0) , 10% GTE fermented by *A. niger* RAF106 for 4 days, and 10% GTE fermented for 8 days.

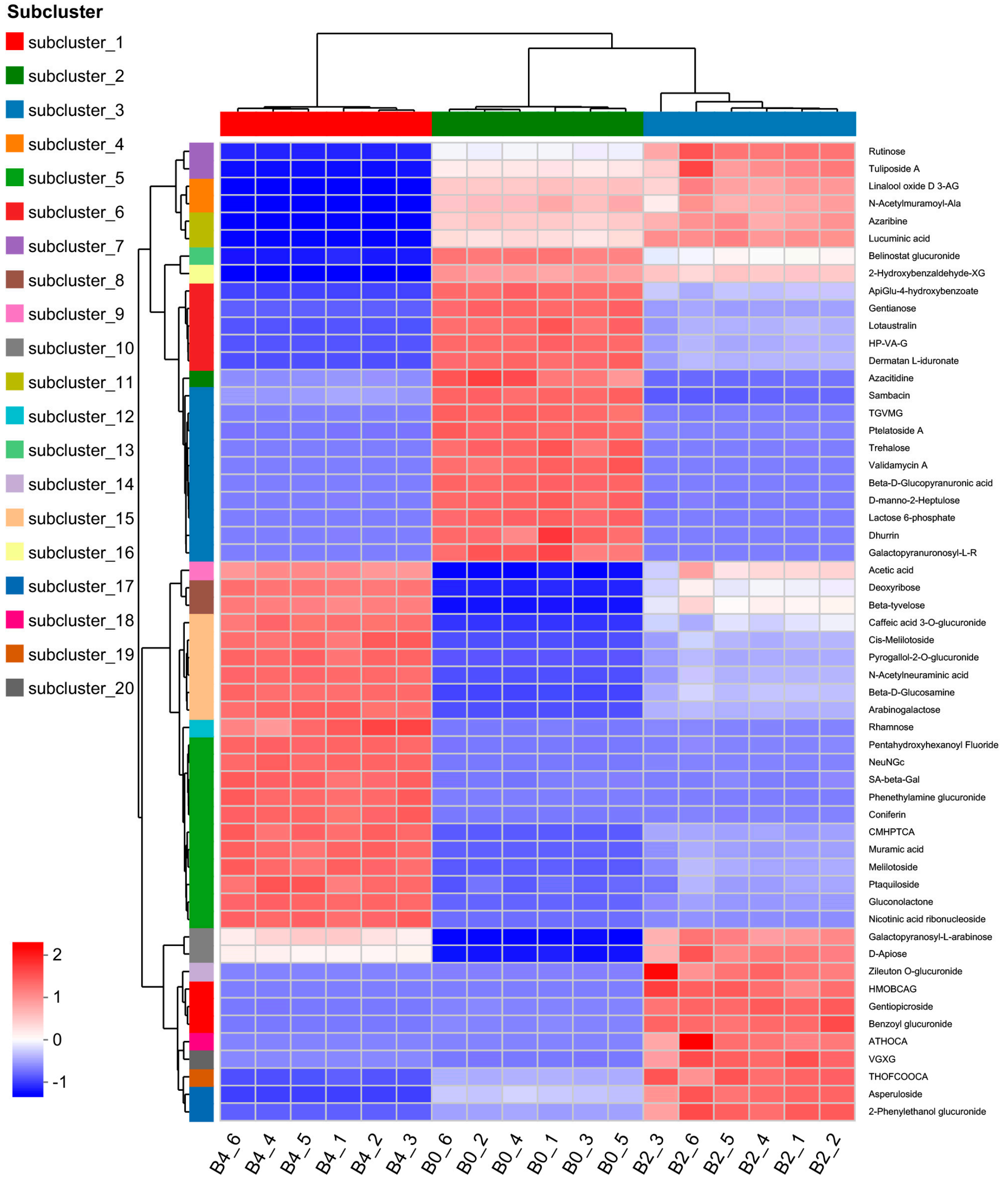


**Fig. S5.** Heatmap analysis of metabolites belonging to carbohydrates and carbohydrate conjugates in different samples. Linalool oxide D 3-AG: Linalool oxide D 3-[apiosyl-(1->6)-glucoside]. 2-Hydroxybenzaldehyde-XG: 2-Hydroxybenzaldehyde O-[xylosyl-(1->6)-glucoside]. ApiGlu-4-hydroxybenzoate: Apiosylglucosyl 4-hydroxybenzoate. HP-VA-G: 4-Hydroxy-5-(phenyl)-valeric acid-O-glucuronide. TGVMG: 5-(3',4',5'-trihydroxyphenyl)-gamma-valerolactone-O-methyl-4'-O-glucuronide.

Galactopyranuronosyl-L-R: 2-O-a-D-Galactopyranuronosyl-L-rhamnose. CMHPTCA: 6-[5-(carboxymethyl)-2-hydroxyphenoxy]-3,4,5-trihydroxyoxane-2-carboxylic acid. HMOBCAG: 7-Hydroxy-2-methyl-4-oxo-4H-1-benzopyran-5-carboxylic acid 7-glucoside. ATHOCA: (2S,3S,4S,5R)-6-(4-Acetamidophenoxy)-3,4,5-trihydroxyoxane-2-carboxylic acid. VGXG:Vomifoliol 9-[glucosyl-(1->4)-xylosyl-(1->6)-glucoside]. THOFCOOCA: 3,4,5-trihydroxy-6-({7-oxo-7H-furo[3,2-g]chromen-4-yl}oxy)oxane-2-carboxylic acid. Samples B0, B2, and B4 represented 10% GTE (B0) , 10% GTE fermented by *A. niger* RAF106 for 4 days, and 10% GTE fermented for 8 days.

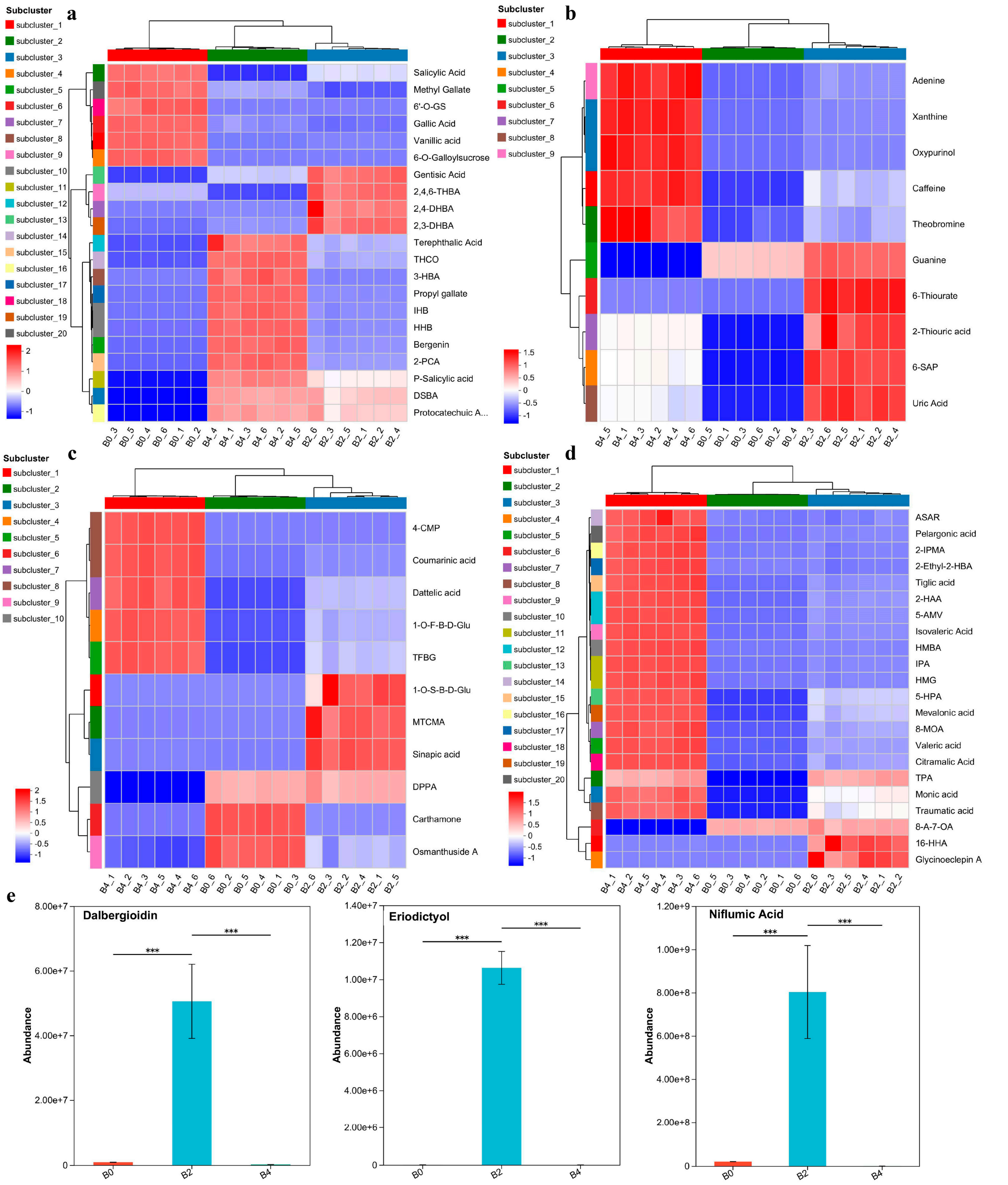


**Fig. S6.** Heatmap analysis of metabolites belonging to benzoic acids and derivatives (a), purines and purine derivatives (b), hydroxycinamic acids and derivatives (c), and fatty acids and conjugates (d) and relative content of dalbergloidin, eriodictyol, and niflumic acid (e) in different samples. 6'-O-GS: 6'-O-Galloylsucrose. 2,4,6-THBA: 2,4,6-Trihydroxybenzoic acid. 2,4-DHBA: 2,4-Dihydroxybenzoic acid. 2,3-DHBA: 2,3-Dihydroxybenzoic acid. THCO:

4R,5R,6S-Trihydroxy-2-hydroxymethyl-2-cyclohexen-1-one 6-(2-hydroxy-6-methylbenzoate). 3-HBA: 3-Hydroxybenzoic acid. IHB: Isobutyl 4-hydroxybenzoate. HHB: Heptyl 4-hydroxybenzoate. 2-PCA: 2-Pyrocatechuic acid. DSBA: 3,5-dihydroxy-4-(sulfooxy)benzoic acid. 6-SAP: 6-Succinoaminopurine. 4-CMP: 4-Coumaroylputrescine. 1-O-S-B-D-Glu: 1-O-Sinapoyl-beta-D-glucose. 1-O-F-B-D-Glu: 1-O-Feruloyl-beta-D-glucose. TFBG: Trans-p-Feruloyl-beta-D-glucopyranoside. MTCMA: Mono-trans-p-coumaroylmesotartaric acid. DPPA: 3-(2,3-Dihydroxyphenyl)-2-propenoic acid. ASAR: Acrylamide-sodium acrylate resin. 2-IPMA: 2-Isopropylmalic acid. 2-Ethyl-2-HBA: 2-Ethyl-2-Hydroxybutyric acid. 2-HAA: 2-Hydroxyadipic acid. 5-AMV: 5-Acetamidovalerate. HMBA: 2-Hydroxy-3-methylbutyric acid. IPA: Isopropylmaleic acid. HMG: 3-Hydroxy-3-methylglutarate. 5-HPA: 5-Hydroxypentanoic acid. 8-MOA: 8-Mercaptooctanoic acid. TPA: Tetranorprostanedioic acid. 8-A-7-OA: 8-Amino-7-oxononanoic acid. 16-HHA: 16-Hydroxyhexadecanoic acid. Samples B0, B2, and B4 represented 10% GTE (B0) , 10% GTE fermented by *A. niger* RAF106 for 4 days, and 10% GTE fermented for 8 days. ***$p$ <0.0001.
